# Development and Validation of a Ferroptosis-Related Gene Signature for Overall Survival Prediction in Lung Adenocarcinoma

**DOI:** 10.3389/fcell.2021.684259

**Published:** 2021-07-07

**Authors:** Qi Tian, Yan Zhou, Lizhe Zhu, Huan Gao, Jin Yang

**Affiliations:** ^1^Department of Medical Oncology, The First Affiliated Hospital of Xi’an Jiaotong University, Xi’an, China; ^2^Department of Breast Surgery, The First Affiliated Hospital of Xi’an Jiaotong University, Xi’an, China

**Keywords:** lung adenocarcinoma, ferroptosis, prognostic signature, overall survival, cell cycle

## Abstract

**Background:** Ferroptosis is an iron-dependent programmed cell death process. Recent studies have found that ferroptosis inducers hold promising potential in the treatment of lung adenocarcinoma (LUAD). However, the comprehensive analysis about the prognostic value of ferroptosis-related genes in LUAD remains to be elucidated.

**Methods:** The RNA sequencing data and corresponding clinical information were obtained from The Cancer Genome Atlas (TCGA) and Gene Expression Omnibus (GEO) databases. A total of 259 ferroptosis-related genes were extracted from FerrDb website. The ferroptosis-related prognostic signature was developed by least absolute shrinkage and selection operator (LASSO) Cox regression analysis in TCGA LUAD cohort, and then validated by 5 independent GEO cohorts. Gene Ontology (GO), Kyoto Encyclopedia of Genes and Genomes (KEGG), and gene set enrichment analysis (GSEA) were performed to identify the difference in biological processes and functions between different risk groups. The expression levels of core prognostic genes were then verified in LUAD samples by immunohistochemistry (IHC) and erastin-treated LUAD cell lines by real-time polymerase chain reaction (PCR). The potential roles of GPX2 and DDIT4 as ferroptosis drivers in LUAD cell line were further confirmed by *in vitro* experiments.

**Results:** A total of 20 intersecting genes between 70 ferroptosis-related DEGs and 45 potential prognostic genes were obtained for LASSO Cox regression analysis. The ferroptosis-related prognostic signature was developed by 7 core prognostic DEGs, and stratified LUAD patients into two risk groups. Kaplan-Meier analysis showed that the overall survival (OS) of LUAD patients in the high-risk group was significantly worse than that of the low-risk group. External validation of 5 independent GEO cohorts further confirmed that the ferroptosis-related prognostic signature was an ideal biomarker for predicting the survival of LUAD patients. Significant enrichment of fatty acid metabolism and cell cycle-related pathways were found in different risk groups. The expression patterns of 7 core prognostic genes in LUAD and adjacent normal lung tissues were validated by IHC, which was almost consistent with the results from public database. Furthermore, the changes related to cell cycle and ferroptosis after erastin treatment were also validated in LUAD cell lines. In addition, silencing GPX2 or DDIT4 could partially reverse the erastin-induced ferroptosis.

**Conclusion:** In summary, the ferroptosis-related prognostic signature based on 7 core prognostic DEGs indicated superior predictive performance of LUAD patients. Targeting ferroptosis holds potential to be a therapeutic alternative for LUAD.

## Introduction

Lung cancer remains the most prevalent cancer and the leading cause of cancer death worldwide (Siegel et al., 2020). Non-small cell lung cancer (NSCLC) accounts for almost 80% of lung cancer, and lung adenocarcinoma (LUAD) is the predominant histological subtype of NSCLC (Shukla et al., 2017). Despite the clinical application of epithelial growth factor receptor (EGFR)-targeted tyrosine kinase inhibitors (TKIs) and immunotherapy targeting PD-1 or PD-L1 in recent years, the 5-year overall survival (OS) rate for LUAD patients still remains 16% (Miller et al., 2016). Therefore, it is urgent to further discover specific prognostic prediction methods for LUAD patients in order to find new therapeutic targets and improve patients’ survival.

Ferroptosis is an iron-dependent programmed cell death process, which is different from other forms of cell death, such as apoptosis, necrosis and autophagy. The main characteristic of ferroptosis is that it is induced by the imbalance of cellular redox homeostasis, leading to excessive lipid peroxidation and finally resulting in cell death. The ferroptosis-related diseases mainly include neurodegenerative diseases (Ren et al., 2020), organ injury induced by ischemia (Friedmann Angeli et al., 2014), and several types of cancers (Jiang et al., 2020). The rapid growth of studies on the role of ferroptosis in cancer has boosted a perspective for its application in cancer treatment. Recently, Alvarez et al. (2017) reported that inhibition of iron-sulfur cluster biosynthetic enzyme NFS1 in LUAD cooperated with suppression of cysteine transport to trigger ferroptosis *in vitro* and slow tumor growth. Besides, Liu et al. (2020) found that the transcription factor nuclear factor-erythroid 2-like 2 (NRF2) inhibitor (Brusatol) could enhance the sensitivity of NSCLC cells to cystine deprivation-induced ferroptosis by FOCAD-FAK signaling, and the combination use of Brusatol and erastin showed better therapeutic action against NSCLC. Other novel compounds, such as erianin, could exert anti-tumor effects in lung cancer by inducing Ca^2+^/CaM-dependent ferroptosis and inhibiting cell migration (Chen P. et al., 2020). In summary, ferroptosis inducers hold promising potential in the treatment of patients with NSCLC, especially for tumors resistant to traditional treatment. Recent studies have suggested that ferroptosis may play a role in tumor suppression by inducing cell cycle arrest and metabolic regulation imbalance (Li et al., 2020). In addition, almost all of the well-recognized ferroptosis regulators are main components modulating cellular redox (Bersuker et al., 2019; Doll et al., 2019) and cell cycle (Jiang et al., 2015; Ou et al., 2016). However, there is still a lack of comprehensive understanding of ferroptosis-related genes in the prognosis of LUAD, and the function of ferroptosis in LUAD remains largely unknown.

In the present study, we developed a 7-gene ferroptosis-related prognostic signature by analyzing the RNA-seq data and corresponding clinical information of LUAD patients from TCGA database. We further validated our 7-gene model in 5 independent GEO cohorts. Moreover, we detected the expression profiles of core genes in clinical tissue samples. The possible role and functions of core genes in regulating ferroptosis in LUAD cell lines were also explored.

## Materials and Methods

### Patients and Datasets

All public datasets included in this study meet the following inclusion criteria: (1) More than 50 patients were included in each cohort; (2) Included patients had been pathologically confirmed with LUAD; (3) The clinical characteristics of included patients were relatively complete; (4) Included patients had follow-up information of OS. A total of 1163 LUAD samples from six public datasets were included in our study. RNA-Seq expression profile of 594 samples (including 535 LUAD samples and 59 normal lung samples), and the corresponding clinical information of 522 samples were downloaded from the Cancer Genome Atlas (TCGA) database. In addition, the transcriptome and clinical information of 5 external validation cohorts were downloaded from Gene Expression Omnibus (GEO) database, including 90 samples of GSE11969 (Takeuchi et al., 2006), 117 samples of GSE13213 (Tomida et al., 2009), 83 samples of GSE30219 (Rousseaux et al., 2013), 158 samples of GSE31210 (Okayama et al., 2012), and 180 samples of GSE41271 (Sato et al., 2013). The main demographic and clinical characteristics of LUAD samples in the datasets mentioned above were summarized in Table 1. Patients with survival time less than 90 days were excluded for Cox and Kaplan-Meier analysis.

**TABLE 1:** Main demographic and clinical characteristics of LUAD patients in different datasets.

Characteristics	TCGA	GSE11969	GSE13213	GSE30219	GSE31210	GSE41271	IHC cohort
Number	522	90	117	85	158	184	30
Age, median (range)	66 (33–88)	62 (32–84)	61 (32–84)	60 (44–84)	60.5 (30–75)	−	63 (38–83)
Gender (%)							
Female	280, 53.6%	43, 47.8%	57, 48.7%	19, 22.4%	94, 59.5%	91, 49.5%	13, 43.3%
Male	242, 46.4%	47, 52.2%	60, 51.3%	66, 77.6%	64, 40.5%	93, 50.5%	17, 56.7%
TNM stage							
I	279, 53.4%	52, 57.8%	79, 67.5%	84, 98.8%	120, 75.9%	101, 54.9%	13, 43.3%
II	124, 23.8%	13, 14.4%	13, 11.1%		38, 24.1%	29, 15.8%	11, 36.7%
III	85, 16.3%	25, 27.8%	25, 21.4%	1, 1.2%	0	50, 27.2%	6, 20.0%
IV	26, 5.0%	0	0	0	0	4, 2.2%	0
Smoking history							
Yes	446, 85.4%	45, 50.0%	61, 52.1%	−	74, 46.8%	158, 85.9%	12, 40.0%
No	76, 14.6%	45, 50.0%	56, 47.9%	−	84, 53.2%	25, 13.6%	18, 60.0%
Survival status							
OS days (median)	661	2344.5	2,041	2,040	1825.5	1176.5	NA
Censored (%)	188, 36.0%	40, 44.4%	49, 41.9%	45, 52.9%	19, 12.0%	72, 39.1%	NA

### Ferroptosis-Related Genes Definition

259 ferroptosis-related genes were obtained from the FerrDb website^1^, the first database of ferroptosis regulators and markers and ferroptosis-disease associations, which classified ferroptosis-related genes into 3 subgroups, including drivers, suppressors and markers (Zhou and Bao, 2020). After removing the duplicated genes of the above three subgroups of ferroptosis gene sets, we obtained a total of 259 genes for the subsequent analysis. The detailed information and classification of ferroptosis-related genes were attached in Supplementary Table 1.

### Identification of Intersecting Genes to Build the Prognostic Signature

Ferroptosis-related differentially expressed genes (DEGs) between LUAD and adjacent normal lung samples were obtained by R “limma” package, filtered by the cutoff values of false discovery rate (FDR) < 0.05 and log2| fold change (FC)| > 1. The potential ferroptosis-related prognostic genes were selected by univariate Cox analysis by R “survival” filtered by *p* < 0.05. The intersect genes between ferroptosis-related DEGs and prognostic genes were obtained and showed via R “venn” package.

### Development of the Prognostic Signature of Ferroptosis-Related Genes

The intersecting genes between ferroptosis-related DEGs and potential prognostic genes were used as candidates for the construction of the prognostic signature. LASSO (least absolute shrinkage and selection operator)-Cox regression analysis was applied to identify the core prognostic DEGs by the R “glmnet” package, and the value of the penalty parameter (λ) was determined according to the lowest partial likelihood deviance by 10-fold cross-validation. The ferroptosis-related risk scores for each patient was calculated by the following formula:

Risk score = β1^∗^expG1 + β2^∗^expG2 + … +βn^∗^expGn,

Where β is the regression coefficient obtained through LASSO-Cox regression, and expG is the expression level of core prognostic genes. The LUAD samples were divided into low-risk and high-risk groups according to the median value of risk scores. Subsequently, principal component analysis (PCA) by “prcomp” function and t-distributed stochastic neighbor embedding (t-SNE) algorithm by “Rtsne” package were applied to dimensionality reduction analysis between the two risk groups.

### Prognostic Meta-Analysis

To evaluate the efficacy of the prognostic signature based on ferroptosis-related genes in predicting survival of LUAD patients in different public cohorts, a meta-analysis of HR values was performed by random-effects model by STATA 15.0 software.

### Functional Enrichment Analysis

DEGs between high-risk and low-risk groups were identified by the cutoff value of log2| FC| > 1 and FDR < 0.05, and then the Gene Ontology (GO) and Kyoto Encyclopedia of Genes and Genomes (KEGG) analyses based on DEGs were conducted by “clusterProfiler” R package. Gene set enrichment analysis (GSEA) was performed using GSEA4.1.0 software^2^. The c2.cp.kegg.v7.0.symbols.gmt in MSigDB was used as the reference gene sets in GSEA.

### Cell Culture

A549 and H1299 cells were obtained from Beijing union medical college hospital cell resource sharing platform. A549 and H1299 cells were cultured in RPMI 1640 medium with 10% fetal bovine serum (FBS) and 1% Penicillin-Streptomycin, and maintained in a humidified incubator at 37°C, 5% CO_2_. Medium, FBS and Penicillin-Streptomycin were purchased from Corning.

### Cell Viability Assay

Cell Counting Kit-8 (CCK-8) (TargetMol) assay was performed to measure cell viability according to the manufacturer’s instructions. Erastin (T-1765) was purchased from TargetMol. A549 and H1299 cells were seeded in 96-well plates at a density of 3,000 cells per well and incubated in a humidified cell incubator at 37°C, 5% CO_2_ for 24 h. The cells were then treated with erastin of 0, 10, and 20 for 24, 48, and 72 h. The WST-8 reagent was then added and the 96-well plate was continued to incubate for another 2 h. The OD (optical density) values were measured at 450 nm with a microplate reader. The experiments in all groups were performed in triplicates and repeated 3 times.

### Detection of Lipid Peroxidation

A549 and H1299 cells were seeded in a 6-well plate at a density of 1 × 10^6^ cells per well and incubated for 24 h, and then treated with erastin (0, 10, and 20 μM) for another 48 h. Then the cells were harvested by trypsinization and resuspended in 500 μL of PBS containing the BODIPY 581/591 C11 dye (Invitrogen, D3861). Cells were then incubated for 30 min at 37°C and analyzed using a flow cytometer.

### Determination of Iron Concentration

A549 and H1299 cells were seeded in a 6-well plate at a density of 1 × 10^6^ cells per well and treated with erastin (0, 10, and 20μM) for 48 h. Cells were then washed and lysed. The iron concentration was determined with an iron assay kit (Abcam, ab83366) according to the manufacturer’s instructions.

### RNA Isolation and Real-Time PCR

Total RNA from A549 and H1299 cells treated with erastin was extracted using TRIzol reagent (Invitrogen), and then converted to cDNA using the PrimeScript^TM^ RT Master Mix (Takara) in accordance with the manufacturer’s instructions. Real-time PCR was conducted with the SYBR-Green kit (Takara) to detect the mRNA expression levels of core prognostic genes. The primers used for real-time PCR were listed in Supplementary Table 2 and purchased from Sangon Biotech.

### IHC

The samples used for IHC staining had to meet the following inclusion criteria: (1) Age > 18; (2) All included patients had been pathologically confirmed with LUAD; (3) The patients included had no other serious systemic comorbidities; (4) No other primary tumors were found in patients; (5) Good patients’ compliance to continue follow-up. A total of 30 cases of LUAD and 12 cases of adjacent non-cancerous lung tissues were obtained from the First Affiliated Hospital of Xi’an Jiaotong University. Our study was approved by the Ethics Committee on Human Research of the First Affiliated Hospital of Xi’an Jiaotong University. Primary antibodies used in IHC including ALOX12B (ab121785, 1:50 for IHC), ALOX15 (ab244205, 1:200 for IHC), GPX2 (ab140130, 1:200 for IHC), DDIT4 (ab191871, 1:200 for IHC), GDF15 (ab180929, 1:200 for IHC), SLC2A1 (ab115730, 1:500 for IHC), and RRM2 (ab172476, 1:100 for IHC) were purchased from Abcam. The paraffin-embedded tissue sections were roasted at 60°C for 6 h and deparaffinized in xylene and dehydrated in gradient concentration of ethanol. Antigen repair of tissue slides was subsequently performed in sodium citrate buffer (PH = 9.0) in a microwave oven. The endogenous peroxidase was then deactivated by 3% hydrogen peroxide for 15 min. Subsequently, 5% BSA (bovine serum albumin) was applied to block non-specific antigens at room temperature for 30 min and the incubation with primary antibodies in a certain concentration was performed overnight at 4°C. On the second day, the slides were incubated with the homologous secondary antibody at room temperature for 1 h. Then, DAB (diaminobenzidine) staining, hematoxylin staining, dehydration in gradient ethanol and transparency in xylene were followed to handle the slides. 10 random fields of each tissue section were selected for semi-quantitative scoring, and the scoring method was as follows: (1) Positive cell rate score—0 for < 10% positive cells, 1 for 10∼25% positive cells, 2 for 25∼50% positive cells, 3 for 50∼75% positive cells and 4 for > 75% positive cells; (2) Dyeing strength score—1 for light yellow, 2 for brown yellow and 3 for tan; (3) The total score was the product of the positive cell rate score and staining intensity score. The Ki67 and TNM stage information of all samples were obtained from the postoperative pathological reports of the Department of Pathology in our hospital. All LUAD samples were divided into Ki67-low and Ki67-high two groups according to the median value of Ki67 (35%, range from 5 to 60%).

### Small Interfering RNA (siRNA) Transfection

The siRNAs targeting GPX2 and DDIT4 were synthesized from GenePharma and transfected with Lipofectamine 2000 reagent (Invitrogen) according to the manufacturer’s protocol. The knockdown efficiency was evaluated by Western blotting after 48h transfection. The sequences of siRNAs are the following: si-Ctrl: 5′-UUCUCCGAACGUGUCACGUTT-3′; si-GPX2-1: 5′-GCUAGAAGAGACC-AAUAAAGG-3′; si-GPX2-2: 5′-GGGAGAAGGUAGAUUUCAAUA-3′; si-DDIT4-1: 5′-GUACU-GUAGCAUGAAACAAAG-3′; and si-DDIT4-2: 5′-GAGGAGUGUUGAACUUCAACC-3′. The siRNA sequences used in cell viability assay and iron concentration detection were siGPX2#1 and siDDI4#1.

### Western Blotting

Whole cell lysates were prepared by RIPA buffer, then proteins were separated with SDS/PAGE gel and transferred to PVDF membranes, which were incubated overnight with corresponding primary antibodies. Primary antibodies used in Western blotting included CDK4 (#12790 from CST, 1:1,000), CDK6 (#3136 from CST, 1:2,000), p21 (#2947 from CST, 1:1,000), p27 (#3686 from CST, 1:1,000), GPX2 (ab140130 from abcam, 1:5,000), DDIT4 (ab191871 from abcam, 1:1,000), and HRP-labeled GAPDH (HRP-60004 from proteintech, 1:15,000). Subsequently, HRP-conjugated secondary antibodies (Cell Signaling Technology) were incubated and chemiluminescent signals were detected by ECL (Millipore).

### Statistical Analysis

All data were analyzed using R version 3.5.2 or Graphpad Prism 8, and all experiments were repeated at least 3 times. These results were presented as mean ± standard deviation (SD). Student’s two-sided *t*-test was used to compare the differences between two groups. Differences in survival between different risk groups were compared by Kaplan-Meier curves followed by log-rank test. *P* < 0.05 was considered as statistically significant.

## Results

**FIGURE 1:**
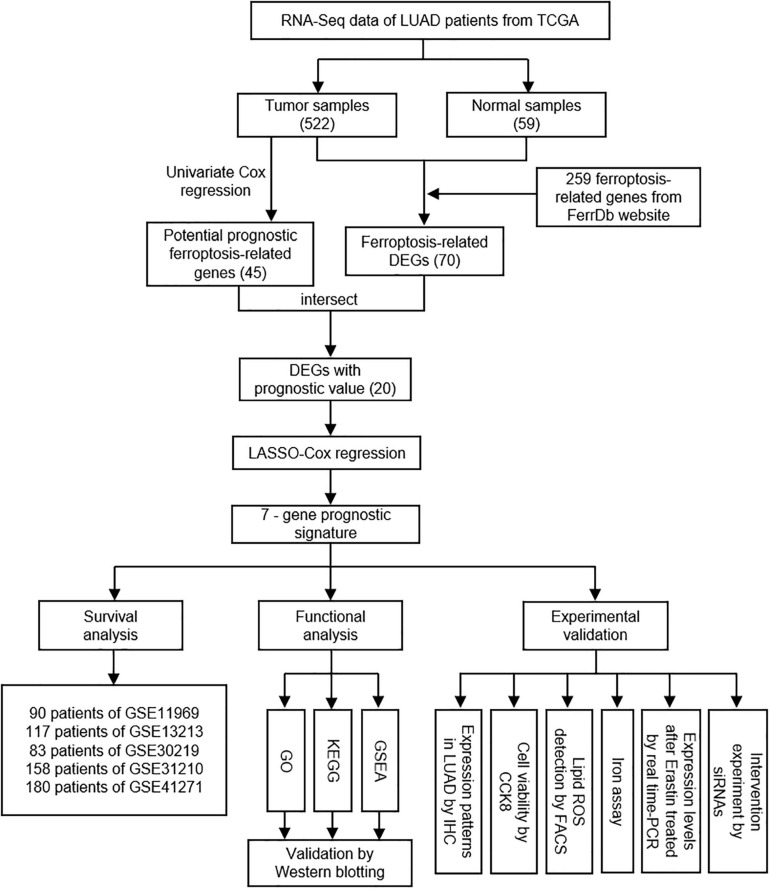
The overall study design and workflow.

The overall design and flow chart of this study were shown in Figure 1. 522 LUAD patients in TCGA database and 628 LUAD patients in GEO database were included in this study. The main demographic and clinical characteristics of patients in different datasets were summarized in Table 1.

### The Prognostic Significance of Ferroptosis-Related Genes in LUAD

A total of 259 well-defined ferroptosis-related genes were included in this study, including 108 drivers that promote ferroptosis, 69 suppressors that prevent ferroptosis, and 111 markers that indicate the occurrence of ferroptosis. There is an overlap between these three gene sets. The detailed ferroptosis-related gene sets could be found in Supplementary Table 1. The univariate Cox proportional hazards regression analysis was applied to assess the relationship between the expression levels of ferroptosis-related genes and prognosis of LUAD patients of TCGA database. A total of 45 potential prognostic ferroptosis-related genes were identified (*p*-value < 0.05), and the prognostic information of these 45 genes was shown in Table 2. Among these 45 genes, 15 genes were identified as “protective” factors with hazard ratios (HRs) < 1, while the remaining 30 genes were considered as “risk” factors with HRs > 1.

**TABLE 2:** Univariate Cox analysis of ferroptosis-related genes in TCGA cohort.

Official symbol	Classification	HR	HR.95L	HR.95H	*p*-value
SLC7A11	Suppressor, marker	1.1436	1.0264	1.2741	0.0150
GCLC	Suppressor	1.1311	1.0274	1.2453	0.0120
SLC3A2	Suppressor, marker	1.2903	1.0164	1.6379	0.0363
CISD1	Suppressor	1.4746	1.0815	2.0106	0.0141
FANCD2	Suppressor	1.4818	1.1262	1.9496	0.0050
HELLS	Suppressor	1.2736	1.0203	1.5899	0.0326
VDAC2	Driver, suppressor	1.5990	1.2089	2.1150	0.0010
CISD2	Suppressor	1.4245	1.0326	1.9651	0.0311
ISCU	Suppressor	0.5316	0.3645	0.7753	0.0010
ACSL3	Suppressor	1.4123	1.1310	1.7635	0.0023
ARNTL	Suppressor	0.6487	0.4691	0.8971	0.0089
ATP5MC3	Driver, marker	1.3624	1.0458	1.7748	0.0219
DUOX1	Driver	0.8690	0.7636	0.9891	0.0335
PGD	Driver	1.1832	1.0226	1.3691	0.0238
FLT3	Driver	0.4923	0.2676	0.9058	0.0227
NRAS	Driver	1.3643	1.0743	1.7327	0.0108
KRAS	Driver	1.3267	1.0652	1.6524	0.0116
GLS2	Driver	0.3929	0.1930	0.8000	0.0100
ALOX12B	Driver	1.5515	1.0374	2.3206	0.0325
ALOX15	Driver, marker	0.8494	0.7296	0.9890	0.0355
ALOX15B	Driver	0.9102	0.8330	0.9946	0.0375
ALOXE3	Driver	1.6274	1.0031	2.6403	0.0486
PHKG2	Driver	0.7183	0.5258	0.9814	0.0377
DPP4	Driver	0.9036	0.8279	0.9863	0.0233
PEBP1	Driver	0.6239	0.4811	0.8091	0.0004
CDO1	Driver	0.7163	0.5199	0.9870	0.0413
PANX1	Driver	1.3978	1.0663	1.8323	0.0153
UBC	Marker	1.4774	1.0163	2.1477	0.0409
TXNRD1	Marker	1.1429	1.0354	1.2615	0.0081
SRXN1	Marker	1.1887	1.0130	1.3949	0.0341
GPX2	Marker	1.0511	1.0004	1.1043	0.0481
DDIT4	Marker	1.2211	1.0740	1.3883	0.0023
TSC22D3	Marker	0.8428	0.7269	0.9771	0.0234
SLC7A5	Marker	1.1724	1.0379	1.3244	0.0105
HERPUD1	Marker	0.6625	0.5232	0.8389	0.0006
GDF15	Marker	0.8923	0.8103	0.9825	0.0204
CEBPG	Marker	1.3520	1.0752	1.6999	0.0099
EIF2S1	Marker	1.7254	1.2530	2.3757	0.0008
RELA	Marker	1.6419	1.0522	2.5623	0.0290
IL33	Marker	0.8559	0.7507	0.9758	0.0200
SLC2A1	Marker	1.2901	1.1569	1.4386	0.0000
RRM2	Marker	1.3596	1.1913	1.5517	0.0000
HNF4A	Marker	1.2844	1.1014	1.4978	0.0014
YWHAE	Marker	1.4306	1.0352	1.9769	0.0300
AURKA	Marker	1.3003	1.1259	1.5018	0.0004

### Identification of Prognostic Ferroptosis-Related DEGs in LUAD

**FIGURE 2:**
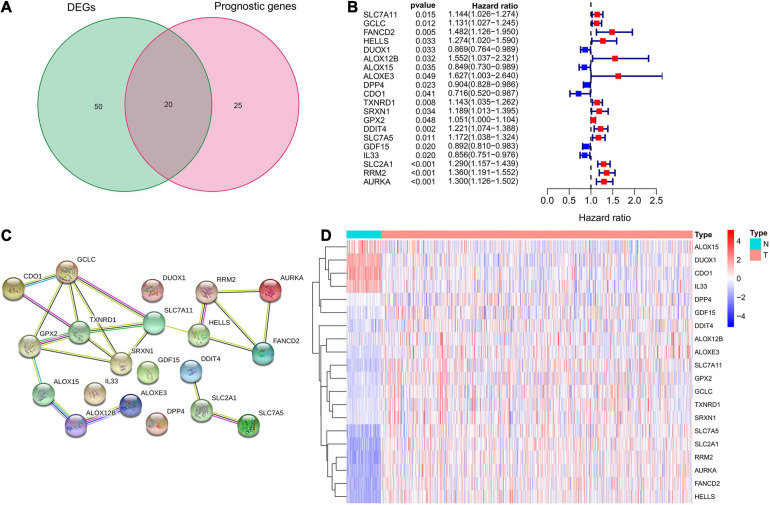
Identification of potential prognostic ferroptosis-related DEGs in LUAD. **(A)** Venn diagram to show the 20 intersection genes of 70 ferroptosis-related DEGs between LUAD and adjacent non-tumorous samples and 45 potential prognostic genes identified by univariate Cox analysis. **(B)** The forest plot showing the results of univariate Cox regression analysis of these 20 ferroptosis-related genes. **(C)** The protein interaction network among these 20 genes from the STRING database. **(D)** The heatmap showing the expression levels of these 20 genes in LUAD and normal lung tissues. DEGs, differentially expressed genes; LUAD, lung adenocarcinoma.

A total of 70 ferroptosis-related DEGs between LUAD and adjacent non-tumorous samples of TCGA database were identified by the criteria of log2| FC| > 1, FDR < 0.05. The heatmap and volcano map in Supplementary Figure 1 depicted the expression level and distribution of these ferroptosis-related DEGs, including 47 DEGs up-regulated and 23 DEGs down-regulated in LUAD. Subsequently, 20 intersection genes of 70 ferroptosis-related DEGs and 45 potential prognostic genes were obtained as the potential prognostic ferroptosis-related DEGs, which was shown in the venn diagram of Figure 2A. The forest plot of Figure 2B showed the results of univariate COX regression analysis of these 20 genes, which demonstrated that 6 of these genes (DUOX1, ALOX15, DPP4, CDO1, GDF15, IL33) were “protective” factors for LUAD patients, while the other 14 genes were “risk” factors. The protein interaction network among these genes from the STRING database was shown in Figure 2C. The heatmap in Figure 2D showed the expression levels of these 20-potential prognostic ferroptosis-related DEGs in LUAD and normal lung tissues, from which we could found that only 4 genes (ALOX15, DUOX1, CDO1, and IL33) were highly expressed in normal tissues, while the remaining 16 genes were highly expressed in tumor tissues. The boxplot in Supplementary Figure 1C also detected the difference in expression levels of these 20 genes in tumor and normal tissues.

### Development of the Ferroptosis-Related Prognostic Signature in TCGA Cohort

**FIGURE 3:**
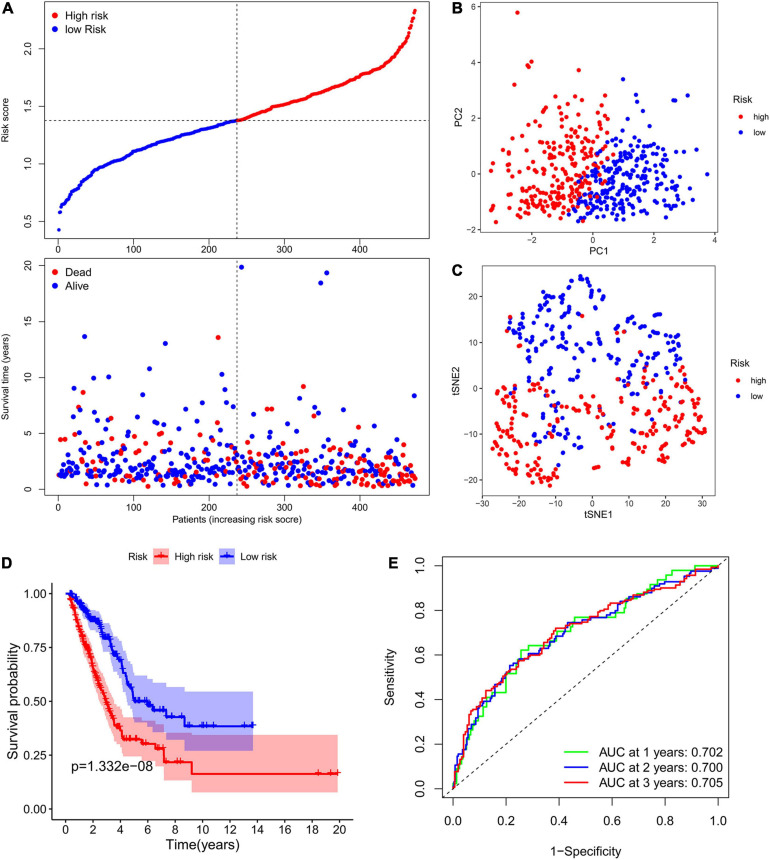
Construction of the ferroptosis-related prognostic signature in the TCGA cohort. **(A)** Distribution of risk scores and survival status in TCGA cohort. **(B,C)** Dimensionality reduction algorithms of PCA **(B)** and t-SNE **(C)** to show the samples of different ferroptosis-related risk groups were separately distributed. **(D)** Kaplan-Meier survival curve showing the OS of LUAD patients in the high-risk group was significantly worse than that of the low-risk group. **(E)** AUC of time-dependent ROC curves to evaluate the predictive efficacy of the prognostic signature for OS in LUAD patients. TCGA, the Cancer Genome Atlas; PCA, principal component analysis; t-SNE, t-distributed stochastic neighbor embedding algorithm; OS, overall survival; AUC, area under the curve; ROC, receiver operating characteristic curve.

The expression levels of the above mentioned 20 potential prognostic ferroptosis-related DEGs and the OS data of LUAD patients with survival time greater than 90 days were then extracted from TCGA database for LASSO Cox regression analysis. The prognostic signature based on 7 core prognostic ferroptosis-related DEGs was then constructed by the optimal penalty parameter (λ) for the LASSO model was determined via 10-fold cross-validation. The risk scores for this model can be obtained by the following formula: Risk score = 0.139 ^∗^ expression level of ALOX12B + (−0.033)^∗^ expression level of ALOX15 + 0.029 ^∗^ expression level of GPX2 + 0.089 ^∗^ expression level of DDIT4 + (−0.021) ^∗^ expression level of GDF15 + 0.088 ^∗^ expression level of SLC2A1 + 0.150 ^∗^ expression level of RRM2. Subsequently, the LUAD patients were stratified into two different risk groups: high-risk (*n* = 236) and low-risk (*n* = 237) according to the median value of risk scores. Patients in the high-risk group had a higher death probability than those in the low-risk group (Figure 3A). Dimensionality reduction algorithms of PCA and t-SNE were then used to confirm the samples of the above two risk groups were separately distributed (Figures 3B,C). Kaplan-Meier survival curve in Figure 3D showed that OS of LUAD patients in the high-risk group was significantly worse than that of the low-risk group, with the HR value of 2.396 (95% confidence interval: 1.771–3.242, *p* < 0.001). The predictive efficacy of the prognostic signature for OS in LUAD patients was evaluated by the time-dependent ROC curves, and the area under the curve (AUC) reached 0.702 for 1-year, 0.700 for 2-year, and 0.705 for 3-year, respectively (Figure 3E).

### Validation of the Ferroptosis-Related Prognostic Signature in Independent GEO Cohorts

**FIGURE 4:**
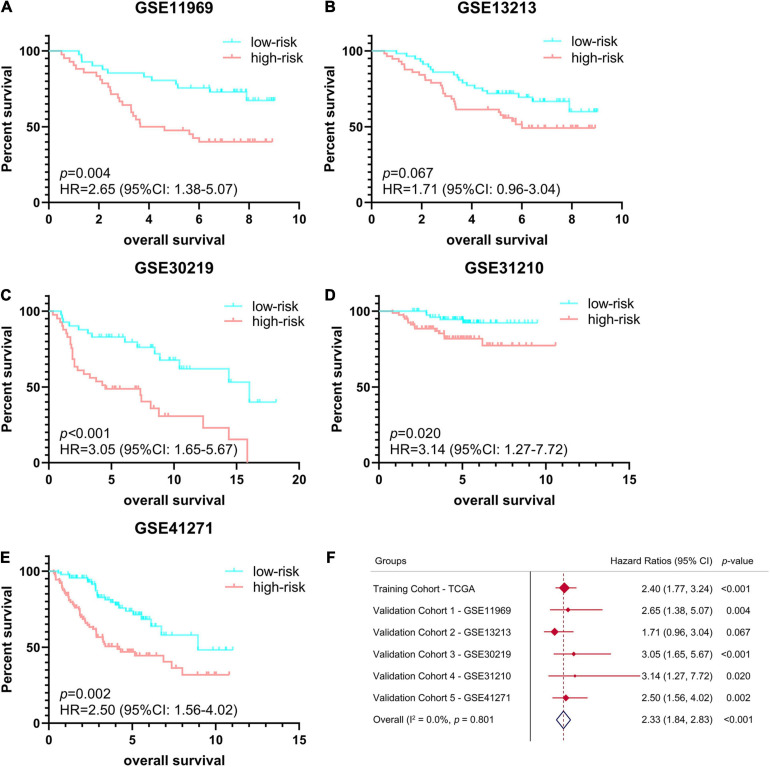
Validation of ferroptosis-related prognostic signature in LUAD from different GEO cohorts. **(A–E)** Kaplan-Meier survival curves of OS in 5 independent GEO cohorts, **(A)** GSE11969 (*n* = 90), **(B)** GSE13213 (*n* = 117), **(C)** GSE30219 (*n* = 85), **(D)** GSE31210 (*n* = 158), **(E)** GSE41271 (*n* = 184). **(F)** Meta-analysis was performed to combine HR values obtained from TCGA and 5 GEO cohorts. GEO, Gene Expression Omnibus; HR, hazard ratio.

In order to verify the stability and reproducibility of the ferroptosis-related prognostic signature of LUAD, 5 independent NSCLC cohorts from the GEO database were used for external validation. The expression levels of ferroptosis-related genes and prognostic information of LUAD patients were obtained from these 5 cohorts. The main demographic and clinical information were provided in Table 1. The formula obtained from the TCGA training cohort was then used to calculate the risk scores of LUAD patients in the GEO validation cohorts. K-M survival analysis showed that the OS of LUAD patients in the high-risk group was significantly worse than that of the low-risk group in 4 GEO validation cohorts, except for the no statistically significant difference found in GSE13213 (Figures 4A–E). Moreover, a meta-analysis was conducted to comprehensively evaluate the HR values obtained from TCGA and GEO cohorts, and the pooled analysis further confirmed that the high-risk group was a risk factor for LUAD patients with combined HR of 2.33 (95% CI: 1.84–2.83, *p* < 0.001) (Figure 4F).

### Independent Prognostic Value of the Ferroptosis-Related Prognostic Signature

**FIGURE 5:**
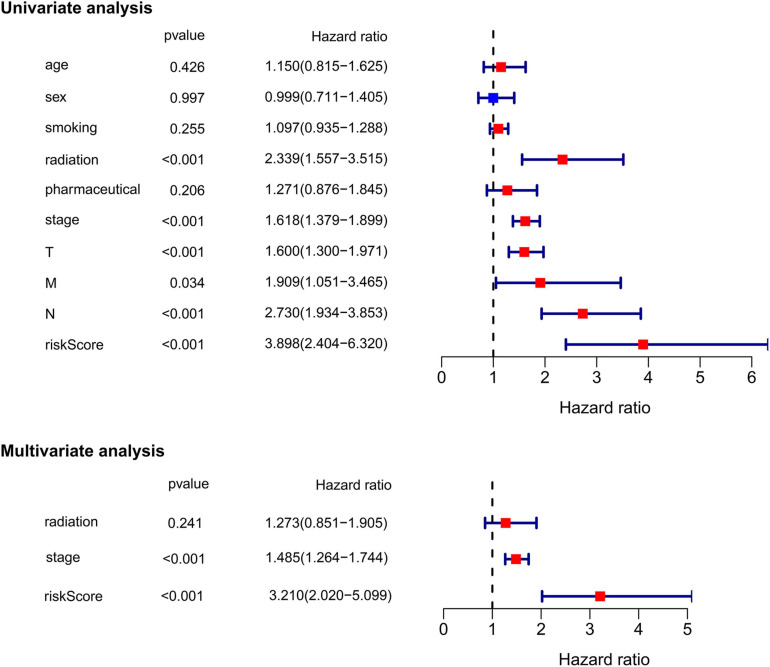
Univariate and multivariate Cox regression analysis of the ferroptosis-related prognostic signature and other clinical characteristics in TCGA cohort.

Subsequently, we extracted the main clinical characteristics of LUAD patients in TCGA database, including age, sex, smoking history, radiation history, pharmaceutical history, T, N, M, and stage. Both univariate and multivariate Cox analyses were carried out among these available variables in combination with risk scores obtained by the ferroptosis-related prognostic signature. The results of univariate Cox analysis confirmed that receiving radiation treatment, higher T/N/M and stage, as well as higher risk scores were risk factors for LUAD patients with HRs > 1, *p* < 0.05 (Figure 5). Multivariate Cox analysis further conducted by including radiation history, stage and risk scores, after other confounding factors were removed, and results showed that higher stage and higher risk scores proved to be independent prognostic factors for OS of LUAD patients (HR for stage = 1.485, 95% CI: 1.264–1.744, *p* < 0.001; HR for risk score = 3.210, 95% CI: 2.020–5.099, *p* < 0.001; Figure 5).

### Identification of the Prognostic Signature-Related Biological Pathways

**FIGURE 6:**
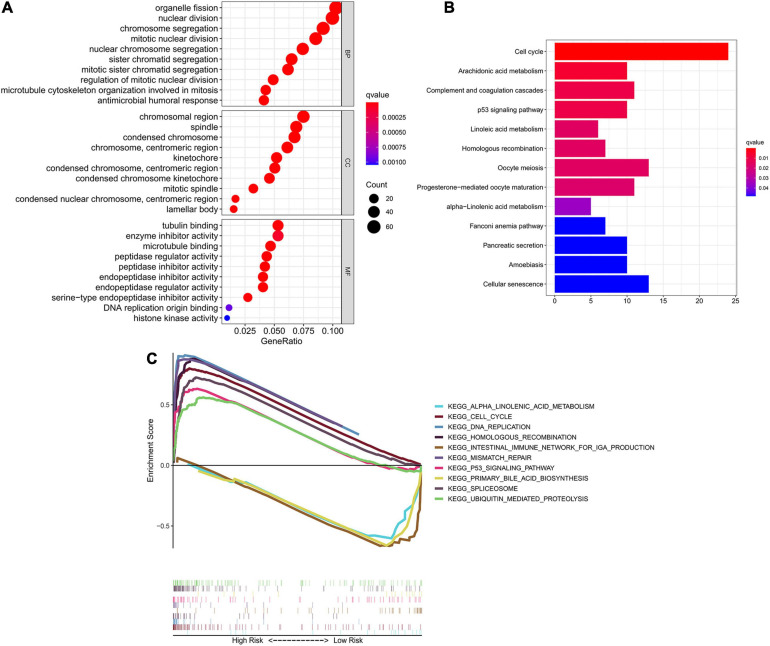
Identification of biological pathways associated with the risk of ferroptosis. **(A)** GO analysis of DEGs between different risk groups into three functional groups, including BP, CC and MF. **(B)** KEGG analysis of DEGs between different risk groups. **(C)** Multiple-GSEA between the high-risk and low-risk groups of TCGA cohort. GO, Gene Ontology; BP, biological process; CC, cellular component; MF, molecular function; KEGG, Kyoto Encyclopedia of Genes and Genomes; GSEA, Gene set enrichment analysis.

To investigate the underlying difference in biological processes and functions between the different risk groups, DEGs between high-risk and low-risk groups were identified by the cutoff value of log2| FC| > 1 and FDR < 0.05, and enrichment analyses of GO and KEGG pathways of DEGs were then performed. Interestingly, DEGs up-regulated in high-risk group were obviously enriched in a variety of cell cycle-related pathways and functions. In Figure 6A, GO analysis showed that the DEGs were significantly enriched in biological process (BP) associated with cell division, including organelle fission, nuclear division, chromosome segregation and etc. For cellular component (CC) item, the up-regulated DEGs were also enriched in chromosome-associated structure (such as chromosomal region, spindle, centromeric region, and kinetochore). The bar plot of Figure 6B showed that the significantly enriched KEGG pathways, including cell cycle, complement and coagulation cascades, p53 signaling, cellular senescence and fatty acid metabolism. Multiple-GSEA analysis also confirmed the enrichment of cell cycle-associated pathways, p53 signaling and metabolic-related pathways (Figure 6C).

### Validation of the Expression Levels of 7 Core Prognostic Genes in LUAD and Paracancerous Normal Lung Tissues

**FIGURE 7:**
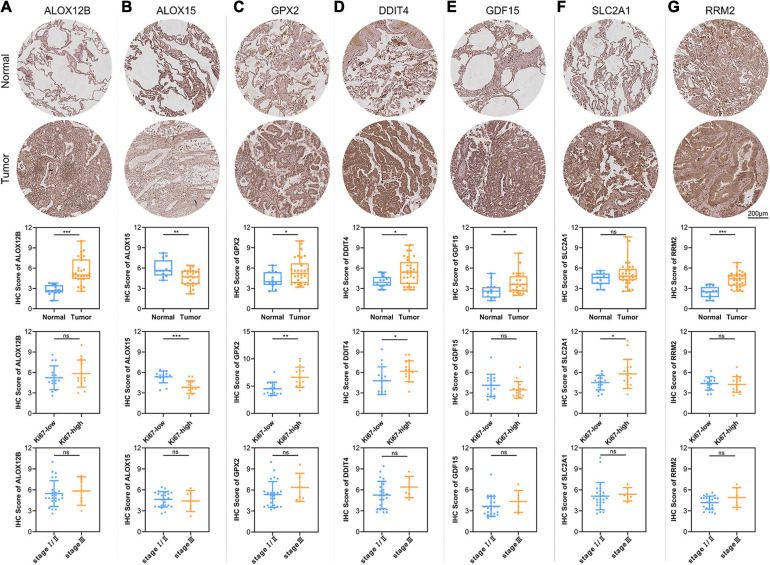
Validation of the expression patterns of 7 core prognostic genes in LUAD tissue samples. The figures of IHC staining showed the representative images of the expression levels of ALOX12B **(A)**, ALOX15 **(B)**, GPX2 **(C)**, DDIT4 **(D)**, GDF15 **(E)**, SLC2A1 **(F)**, and RRM2 **(G)** in adjacent normal lung tissues and LUAD. The box plots showed the statistical results of the expression of the above 7 core prognostic genes in 30 LUAD and 12 adjacent normal lung tissues. The scatter plots showed the distribution of 7 core prognostic genes in LUAD samples with different Ki67 levels and TNM stage. ns, not significant; *P < 0.05; **P < 0.01; ***P < 0.001.

To verify the reliability of the above results obtained from the public database, we collected 30 cases of LUAD samples and 12 cases of adjacent normal lung tissues to test the expression levels of the 7 ferroptosis-related genes consisting of our prognostic signature by IHC. As shown in Figure 7, the expression levels of ALOX12B, GPX2, DDIT4, GDF15, and RRM2 in tumor tissues were significantly higher than those in the normal lung tissues. Only ALOX15 showed high expression in normal lung samples. However, there was no significant difference in the expression level of SLC2A1 between LUAD and adjacent tissues. The above protein expression results of the prognostic genes in clinical tissue samples by IHC were almost consistent with the RNA sequencing data analyzed by the TCGA database. The above 7 prognostic biomarkers were mainly located in the cytoplasm and membrane of LUAD samples with moderate to strong positive staining. Furthermore, we analyzed the relationship between the expression profiles of the 7 core prognostic genes with Ki67 and TNM stage, and found only ALOX15 was significantly low expressed in Ki67-high samples, while GPX2, DDIT4, and SLC2A1 were high expressed in Ki67-high samples. There was no significant difference in the distribution of the other 3 genes in LUAD samples with different Ki67 levels and all 7 genes in samples with different TNM stage.

### Effects of Ferroptosis Inducer Erastin on the Expression Levels of 7 Core Prognostic Genes in LUAD Cell Lines *in vitro*

**FIGURE 8:**
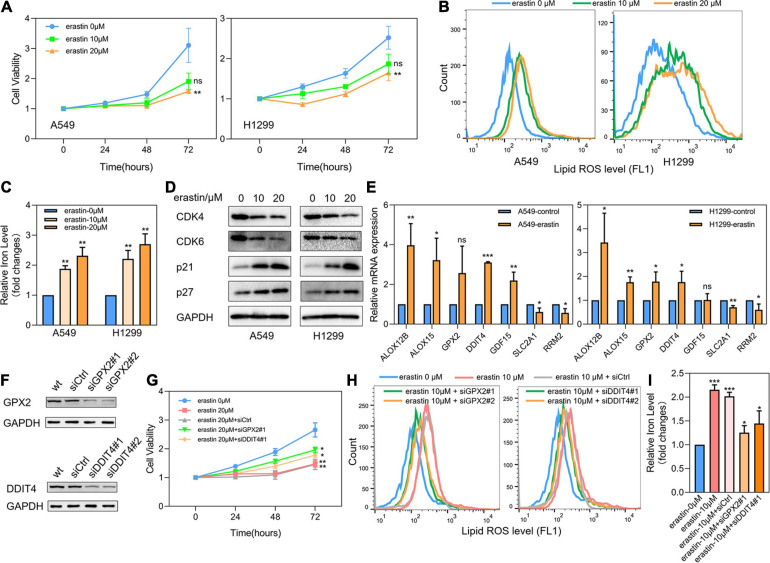
Effects of ferroptosis inducer on expression levels of 7 core prognostic genes in LUAD cell lines *in vitro*. **(A)** The cell viability of A549 and H1299 treated by erastin was tested by CCK8 assay. **(B)** Lipid ROS levels of A549 and H1299 treated by erastin were detected by FACS. **(C)** Iron concentrations of A549 and H1299 treated by erastin were detected by iron colorimetric assay. **(D)** The expression levels of several cell cycle regulators in A549 and H1299 after erastin treatment were assayed by Western blotting. **(E)** The expression changes of 7 core prognostic genes in A549 and H1299 after treated with erastin were detected by real time PCR. **(F)** Silencing of GPX2 and DDIT4 in A549 by siRNAs. **(G)** The cell viability of A549 after erastin treatment in combination with or without GPX2 or DDIT4 silencing were detected by CCK8 assay. **(H)** Lipid ROS levels of A549 after erastin treatment in combination with or without GPX2 or DDIT4 silencing were detected by FACS. **(I)** Iron concentrations of A549 after erastin treatment in combination with or without GPX2 or DDIT4 silencing were detected by iron colorimetric assay. ns, not significant; *P < 0.05; **P < 0.01; ***P < 0.001.

LUAD cell lines A549 and H1299 were then treated with ferroptosis inducer erastin to investigate the role of ferroptosis-related prognostic genes. As shown in Figure 8A, CCK8 assay identified that erastin could inhibit the proliferation of A549 and H1299 in a dose-dependent manner. When the concentration was greater than 20 μM, the growth inhibitory effects of erastin on both A549 and H1299 were statistically significant. Subsequently, we confirmed that erastin treatment of 10 and 20 μM significantly increased the lipid ROS accumulation (Figure 8B) and iron concentration (Figure 8C). Furthermore, to validate the relevance of erastin-induced ferroptosis and cell cycle regulation, we explored the changes in several regulators involved in G1/S transition after erastin treatment of A549 and H1299 cells by Western blotting. As shown in Figure 8D, decreased expression levels of CDK4 and CDK6 were observed, whereas increased expression levels of p21 and p27 were found. Next, real time-PCR was applied to test the expression changes of 7 core prognostic genes in A549 and H1299 after treated with erastin of 20 μM for 48 h. We found that mRNA expression levels of 5 genes (ALOX12B, ALOX15, GPX2, DDIT4, and GDF15) were increased and the other 2 (SLC2A1 and RRM2) were decreased after erastin treatment, although the increase of GPX2 was not statistically significant in A549 and GDF15 was barely changed in H1299 (Figure 8E). To validate the specific role in ferroptosis of individual gene in our prognostic model, silencing GPX2 and DDIT4 were also performed in A549 by siRNAs. Western blotting was used to verify that the expression of GPX2 and DDIT4 would not be affected by the sequence of siCtrl, while two different sequences of siRNAs targeting GPX2 or DDIT4 could effectively reduce the expression of their target genes (Figure 8F). Subsequently, we found that down-regulation of either GPX2 or DDIT4 could partially reverse the cell proliferation arrest (Figure 8G) and the elevation of lipid ROS (Figure 8H) and iron concentration (Figure 8I) induced by erastin in A549 cells, whereas these reversing effects could not be observed in the siCtrl group. The validation of other prognostic genes in our model will be further investigated in our future studies.

## Discussion

Ferroptosis is a novel programmed cell death pattern, mainly caused by iron-dependent lipid peroxidation (Liu et al., 2020). In recent years, the role and mechanism of ferroptosis in different diseases have been extensively investigated, especially in the field of tumor research and treatment. Several studies have already found that some traditional chemotherapy-resistant tumors are highly sensitive to ferroptosis inducers, so ferroptosis is expected to bring novel promising strategies to some refractory tumors (Jiang et al., 2020). In lung cancer, limited ferroptosis-related studies mainly focused on the role of potential ferroptosis biomarkers in the ferroptosis-inducing process of well-recognized inducers (Alvarez et al., 2017; Chen P. et al., 2020; Kwon et al., 2020; Liu et al., 2020; Lou et al., 2020). There is a lack of comprehensive and systematic analysis of ferroptosis in the malignant progression and treatment strategy for lung cancer. In this study, we systematically analyzed the expression profiles and prognostic values of 259 ferroptosis-related genes provided by the latest online FerrDb database (Zhou and Bao, 2020). Additionally, the ferroptosis-related prognostic signature for LUAD patients was developed and validated by available public databases. The cell cycle signaling was identified as the main enrichment pathway between different risk subgroups.

According to the detailed information of ferroptosis-related genes provided by FerrDb website, which was developed by manually extracted 784 ferroptosis-related articles from PubMed, 108 drivers, 69 suppressors, and 111 markers were included in our study as potential prognostic ferroptosis-related genes. Because there are 28 genes reported to function in multiple processes of ferroptosis, these genes were multi-annotated in different subclasses of ferroptosis genes (Zhou and Bao, 2020). Among the 259 ferroptosis-related genes, 27.0% (70/259) were differentially expressed in tumor and adjacent normal tissues of LUAD samples, and 17.3% (45/259) were correlated with prognosis in the univariate Cox analysis. The intersecting genes between ferroptosis-related DEGs and prognostic genes were obtained for the following study. Among the 20 intersecting genes, only 4 were highly expressed in normal lung tissues, and all 4 genes were protective markers according to univariate Cox analysis, which was consistent with the expression status. The remaining 16 genes were highly expressed in tumor tissues of LUAD patients, and most of them (14/16) were identified as risk factors for LUAD patients, except for DPP4 and GDF15. DPP4 (dipeptidyl peptidase 4) is considered as a ferroptosis driver in colorectal cancer. Xie et al. (2017) reported that loss of TP53 could prevent the nuclear translocation of DPP4, thus promoting the plasma membrane-related DPP4-dependent lipid peroxidation process and ultimately leading to ferroptosis, which was consistent with its protective role identified in our analysis. However, study of DPP4 in LUAD showed that it was highly expressed in LUAD compared to adjacent normal lung tissues, and the DPP4 inhibitor could inhibit lung cancer growth by promoting the pro-inflammatory activity of macrophages (Jang et al., 2019). Thus, the specific role of DPP4 in ferroptosis process in LUAD still need to be further studied. According to the study of GDF15 (growth differentiation factor 15) in gastric cancer cell line MGC803, GDF15 functions as a ferroptosis inhibitor in gastric cancer, for knockdown of GDF15 could promote erastin-induced ferroptosis by reducing the expression of SLC7A11 (Chen L. et al., 2020). However, GDF15 was identified as a protective factor for LUAD patients according to the univariate Cox analysis, and how GDF15 functions by ferroptosis process in lung cancer has not been reported.

The prognostic signature developed by LASSO Cox regression was consisted of 7 ferroptosis-related genes, among which the expression of ALOX12B, GPX2, DDIT4, SLC2A1, and RRM2 were positively correlated with the survival risk of LUAD patients, while the levels of ALOX15 and GDF15 were negatively associated with the survival risk. According to FerrDb database, only ALOX12B and ALOX15 were definitely labeled as ferroptosis drivers, while the remaining 5 genes in addition to ALOX15 were labeled as ferroptosis markers, indicating that their definite roles in ferroptosis process remained unclear. After reviewing related references systematically, we summarized the research status in ferroptosis of the above 7 genes included in our prognostic signature as follows: ALOX12B and ALOX15 are two of the six ALOX (arachidonate lipoxygenase) genes in humans. Yang et al. (2016) confirmed that targeted silence of the ALOX family genes by a pool of siRNAs or pharmacological inhibitors in G-401, BJeLR, and HT-1080 cell lines could effectively prevent erastin- or IKE (Imidazole Ketoerastin)-induced ferroptosis, confirming the role of lipoxygenases as ferroptosis drivers. The study by Ou et al. (2016) had found that in various cell lines, including NSCLC H1299, the activation of SAT1 (spermidine N1-acetyltransferase 1) expression induced by TP53 could promote lipid peroxidation and increase the sensitivity to ferroptosis, while PD146176, the specific inhibitor of ALOX15, significantly abrogate the SAT1-induced ferroptosis. Shintoku et al. (2017) also confirmed that, in fibrosarcoma HT1080, pancreatic cancer PANC-1, and NSCLC Calu-1, the cell membrane localized ALOX15 promoted erastin- and RSL3-induced ferroptosis. Glutathione-dependent peroxidases (GPXs) play an important role in catalyzing the reduction reaction of hydrogen peroxide and organic peroxides (Yang et al., 2014), and among them, GPX4 has been identified as a common crucial mediator for ferroptosis, for it reduces hydroperoxyl groups of lipid complexes and inhibits lipoxygenases (Brigelius-Flohe and Flohe, 2020). Also, as a member of GPXs family, GPX2 was proved to control the balance between the regenerative and apoptotic cells in the intestinal epithelium, and restrained the inflammation-induced tumorigenesis in the gut (Brigelius-Flohe and Flohe, 2020). However, its specific role in ferroptosis has not been clarified. DDIT4 (DNA damage inducible transcript 4) was found upregulated by erastin, which was associated with the activation of ER (endoplasmic reticulum) stress response pathway, and the upregulation of CHAC1 (cation transport regulator homolog 1), an ER stress response gene, could be regarded as a marker for pharmacodynamic inhibition of cystine-glutamate exchange (Dixon et al., 2014). Our results also confirmed that knockdown of GPX2 or DDIT4 in A549 could partially reverse the increase of lipid ROS and iron concentration induced by erastin, which further verified the potential role of GPX2 and DDIT4 as ferroptosis drivers. GDF15 (growth differentiation factor 15), as described above, was considered as a ferroptosis inhibitor in gastric cancer (Chen L. et al., 2020), whereas it was upregulated in erastin-treated samples in HT-1080 (Dixon et al., 2014). The above differences may be associated with the heterogeneity and mutant spectrum of different tumors. SLC2A1 (solute carrier family 2 member 1), also known as GLUT1 (glucose transporter 1), plays an important role in glucose transport. Jiang et al. (2017) identified a DNA methylation modifier, LSH (lymphoid-specific helicase), could inhibit ferroptosis in lung cancer by directly transcriptional activation of SLC2A1 expression. RRM2 was reported to be down-regulated in erastin-treated HCC cell lines Bel-7402 and HepG2, and possibly suppress ferroptosis in a GSH-dependent manner (Zhang et al., 2019). In summary, four of the genes (ALOX12B, ALOX15, GPX2, DDIT4) in our prognostic signature have been reported to promote ferroptosis, while SLC2A1 and RRM2 have been regarded as ferroptosis inhibitors, and the role of GDF15 in ferroptosis is not consistent in different tumors.

Although some researchers have investigated the mechanism and clinical application value of ferroptosis, especially in the field of tumor therapy, the underlying mechanism of tumor susceptibility to ferroptosis still needs to be clarified. It is reasonable to find a significant difference in fatty acid metabolism and cell cycle-related pathways in patients with different ferroptosis-related risks. Lipid peroxidation of cell membrane induced by the redox imbalance was the main cause of ferroptosis; thus a significant differential expression of arachidonic acid and linoleic acid metabolism-related genes could be found in different risk groups. Dysregulation of cell cycle-related pathways may be the consequence of ferroptosis imbalance, meanwhile, the abnormal expression of cell cycle-regulating molecules may also lead to different ferroptosis-related risks in LUAD patients. We detected the expression levels of several main cell cycle regulators in A549 and H1299 after treated with erastin, and we found that the positive regulators for cell cycle were down-regulated and the negative regulators were obviously up-regulated. However, the specific modulation relationship between cell cycle regulation and ferroptosis remains to be further elucidated.

Several limitations should be acknowledged in our study: (1) Due to few studies about the role of ferroptosis in tumors, the information of ferroptosis-related genes provided by FerrDb website may not be accurate enough, for the references were manually extracted from the previous literature reports, so some unidentified crucial ferroptosis-mediating genes may be missing in the ferroptosis gene sets. (2) Although all of the 7 genes constituting the ferroptosis-related prognosis signature have been reported to mediating ferroptosis, there is almost no evidence that they could regulate ferroptosis in lung cancer except for ALOX15 and SLC2A1. Therefore, further experimental evidence is needed to validate the ferroptosis-regulating functions of these core prognostic genes in LUAD. 3) A total of 6 cohorts (LUAD cohort of TCGA and 5 GEO cohorts) were used for the construction and external validation of the prognostic signature, however, all the sample information was extracted from the public database. We think this ferroptosis-related prognostic signature would be more reliable if it is tested by a prospective clinical trial cohort in our research center.

## Conclusion

In summary, we systematically analyzed the expression profiles and prognostic significance of 259 ferroptosis-related genes in LUAD patients, and developed a ferroptosis-related prognostic signature consisting of 7 core prognostic DEGs. The efficacy of our prognostic signature was further tested by external validation cohorts. The ferroptosis-related risk was proven to be an independent prognostic factor for LUAD patients. Besides, the expression patterns of 7 core prognostic genes in LUAD and normal lung tissues were confirmed by IHC. The changes related to cell cycle and ferroptosis in LUAD cell line after erastin treatment were also verified by *in vitro* experiments. The potential roles of GPX2 and DDIT4 as ferroptosis drivers in LUAD cell line were further confirmed by siRNA experiments. However, the specific role and regulatory mechanism of these 7 ferroptosis-related genes in LUAD is still needed further experimental verifications. The prospective clinical studies are also needed to validate the clinical application value of our prognostic signature.

## Data Availability Statement

The datasets presented in this study can be found in online repositories. The names of the repository/repositories and accession number(s) can be found in the article/Supplementary Material.

## Ethics Statement

The studies involving human participants were reviewed and approved by the Ethics Committee of the First Affiliated Hospital of Xi’an Jiaotong University. The patients/participants provided their written informed consent to participate in this study.

## Author Contributions

QT and LZ contributed to the study design and performed the experiments. YZ and HG contributed to data collection. QT, YZ, and JY performed statistical analysis and interpretation. QT and JY drafted the manuscript. All authors contributed to critical revision of the final manuscript.

## Conflict of Interest

The authors declare that the research was conducted in the absence of any commercial or financial relationships that could be construed as a potential conflict of interest.

## Footnotes

1 http://www.zhounan.org/ferrdb

2 http://www.broadinstitute.org/gsea/index.jsp

## Supplementary Material

Supplementary Figure S1 Differential expression of ferroptosis-related genes in LUAD and normal lung tissues. The heatmap **(A)** and volcano map **(B)** depicted the expression levels and distribution of 70 ferroptosis-related DEGs. The boxplot **(C)** showed the difference in expression levels of 20 potential prognostic ferroptosis-related DEGs in tumor and normal tissues.

Supplementary Figure S2 Establishment of the prognostic model by LASSO Cox regression. **(A,B)** LASSO regression coefficient profiles. **(C)** Table of regression coefficients of 7 core prognostic genes.
