# Supplementary figures and images for: Development and Validation of a Ferroptosis-Related Gene Signature for Overall Survival Prediction in Lung Adenocarcinoma
Source: Front Cell Dev Biol. 2021 Jul 7;9:684259. doi: 10.3389/fcell.2021.684259 (PMC8294813; doi:10.3389/fcell.2021.684259)

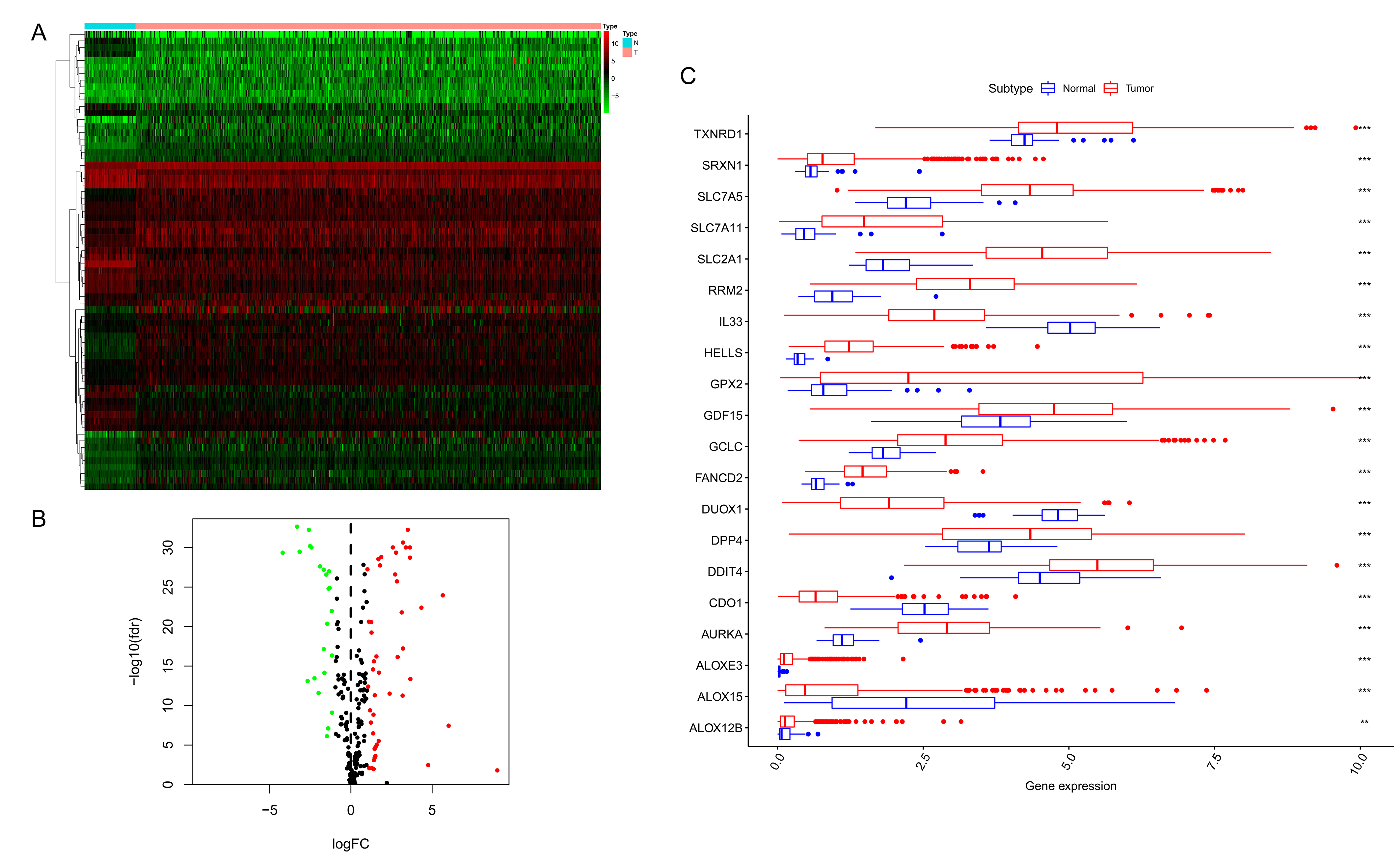

Supplement: Supplementary Figure S1 — Differential expression of ferroptosis-related genes in LUAD and normal lung tissues. The heatmap (A) and volcano map (B) depicted the expression levels and distribution of 70 ferroptosis-related DEGs. The boxplot (C) showed the difference in expression levels of 20 potential prognostic ferroptosis-related DEGs in tumor and normal tissues. [file Image_1.TIF]

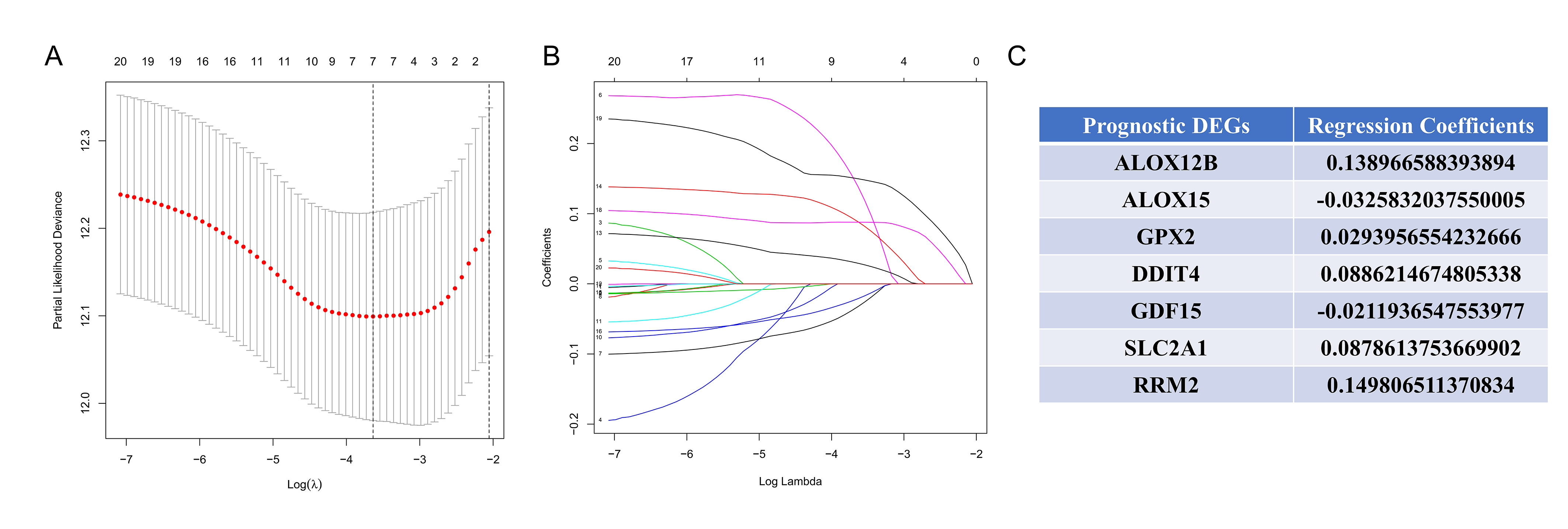

Supplement: Supplementary Figure S2 — Establishment of the prognostic model by LASSO Cox regression. (A,B) LASSO regression coefficient profiles. (C) Table of regression coefficients of 7 core prognostic genes. [file Image_2.TIF]

Figure 8D

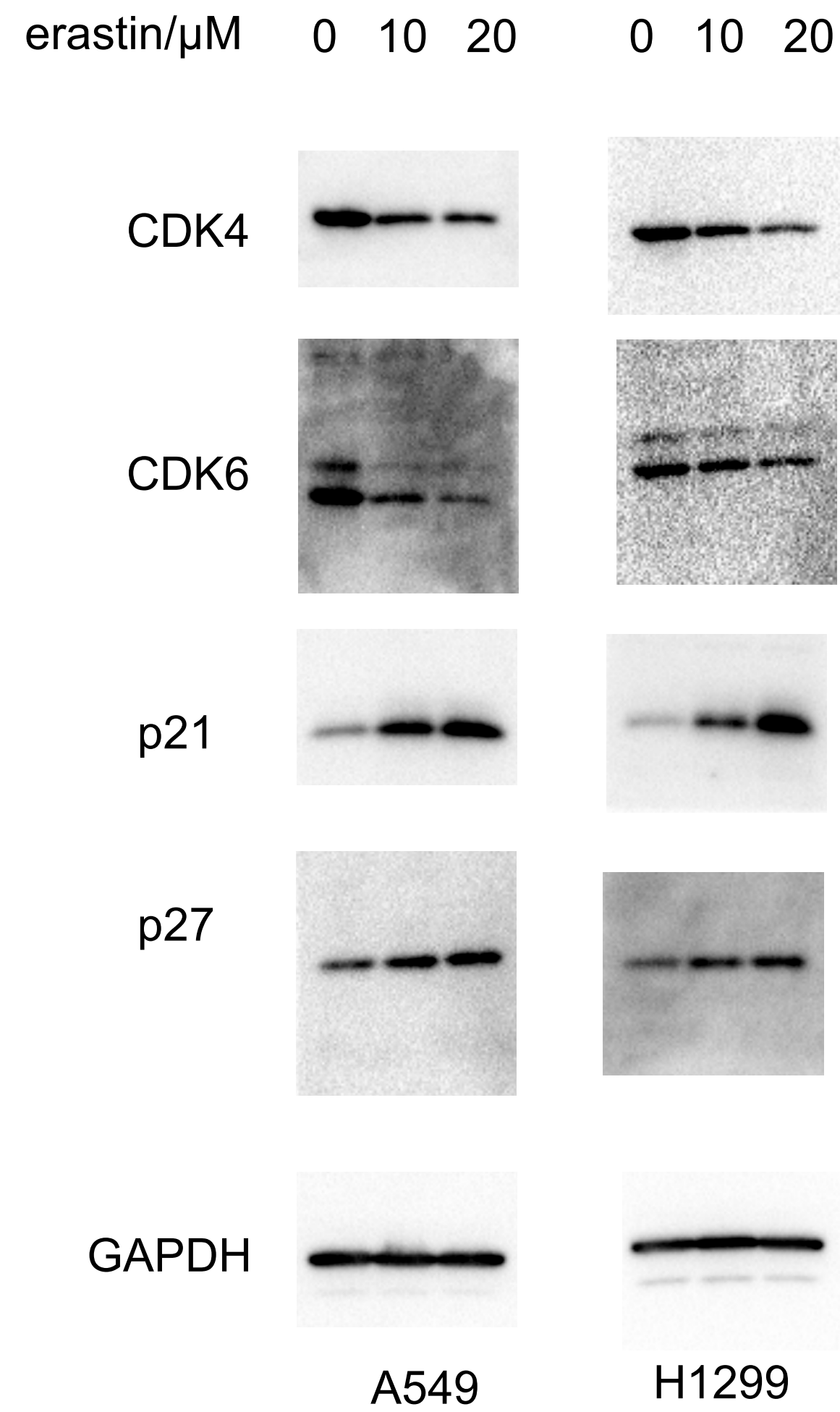

Figure 8F

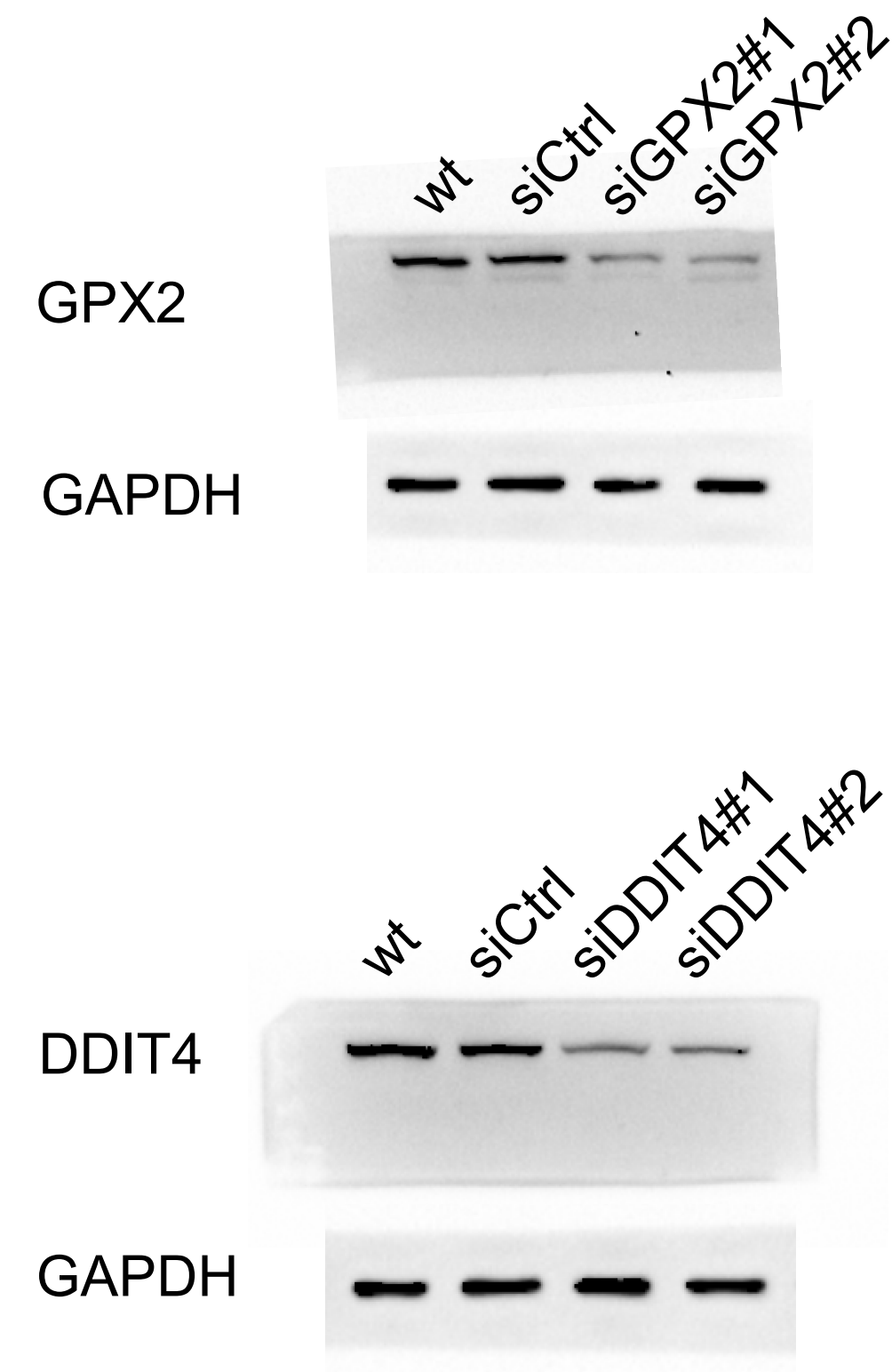

Supplement: Supplementary file 8 [file Data_Sheet_2.PDF]
