# Supplementary material for: Development and Validation of a Ferroptosis-Related Gene Signature for Overall Survival Prediction in Lung Adenocarcinoma
Source: Front Cell Dev Biol. 2021 Jul 7;9:684259. doi: 10.3389/fcell.2021.684259 (PMC8294813; doi:10.3389/fcell.2021.684259)
Supplement: Supplementary file 4 [file Table_2.DOCX]

Supplementary Table 2. The sequences of primers for real-time PCR assays.

| Gene | Sequence |
| --- | --- |
| ALOX12B-F | GCCTGCACAAAGAGCGGTA |
| ALOX12B-R | GGTCTCGTAGCCATCCATCC |
| ALOX15-F | GGGCAAGGAGACAGAACTCAA |
| ALOX15-R | CAGCGGTAACAAGGGAACCT |
| GPX2-F | GGTAGATTTCAATACGTTCCGGG |
| GPX2-R | TGACAGTTCTCCTGATGTCCAAA |
| DDIT4-F | TGAGGATGAACACTTGTGTGC |
| DDIT4-R | CCAACTGGCTAGGCATCAGC |
| GDF15-F | ACCTGCACCTGCGTATCTCT |
| GDF15-R | CGGACGAAGATTCTGCCAG |
| SLC2A1-F | ATTGGCTCCGGTATCGTCAAC |
| SLC2A1-R | GCTCAGATAGGACATCCAGGGTA |
| RRM2-F | CACGGAGCCGAAAACTAAAGC |
| RRM2-R | TCTGCCTTCTTATACATCTGCCA |
